# Identification of a Novel Topoisomerase Inhibitor Effective in Cells Overexpressing Drug Efflux Transporters

**DOI:** 10.1371/journal.pone.0007238

**Published:** 2009-10-02

**Authors:** Walid Fayad, Mårten Fryknäs, Slavica Brnjic, Maria Hägg Olofsson, Rolf Larsson, Stig Linder

**Affiliations:** 1 Department of Oncology and Pathology, Karolinska Institute and Hospital, Stockholm, Sweden; 2 Department of Medical Sciences, Division of Clinical Pharmacology, University Hospital, Uppsala University, Uppsala, Sweden; Roswell Park Cancer Institute, United States of America

## Abstract

**Background:**

Natural product structures have high chemical diversity and are attractive as lead structures for discovery of new drugs. One of the disease areas where natural products are most frequently used as therapeutics is oncology.

**Method and Findings:**

A library of natural products (NCI Natural Product set) was screened for compounds that induce apoptosis of HCT116 colon carcinoma cells using an assay that measures an endogenous caspase-cleavage product. One of the apoptosis-inducing compounds identified in the screen was thaspine (taspine), an alkaloid from the South American tree *Croton lechleri*. The cortex of this tree is used for medicinal purposes by tribes in the Amazonas basin. Thaspine was found to induce conformational activation of the pro-apoptotic proteins Bak and Bax, mitochondrial cytochrome c release and mitochondrial membrane permeabilization in HCT116 cells. Analysis of the gene expression signature of thaspine-treated cells suggested that thaspine is a topoisomerase inhibitor. Inhibition of both topoisomerase I and II was observed using *in vitro* assays, and thaspine was found to have a reduced cytotoxic effect on a cell line with a mutated topoisomerase II enzyme. Interestingly, in contrast to the topoisomerase II inhibitors doxorubicin, etoposide and mitoxantrone, thaspine was cytotoxic to cell lines overexpressing the PgP or MRP drug efflux transporters. We finally show that thaspine induces wide-spread apoptosis in colon carcinoma multicellular spheroids and that apoptosis is induced in two xenograft mouse models *in vivo*.

**Conclusions:**

The alkaloid thaspine from the cortex of *Croton lechleri* is a dual topoisomerase inhibitor effective in cells overexpressing drug efflux transporters and induces wide-spread apoptosis in multicellular spheroids.

## Introduction

The concept of developing target-specific drugs for treatment of cancer has not been as successful as initially envisioned [1], [2]. The success rate of oncology drugs from first-in-man to registration during 1991–2000 was only around 5% for 10 major pharma companies [2]. A major causes of attrition in the clinic is lack of drug efficacy [2]. This realization has lead to a renewed interest in the use of bioassays for drug development in the field of oncology. One attractive screening endpoint is apoptosis since this form of cell death is induced by many clinically used (and effective) anticancer agents [3].

Natural products have been used as source of novel therapeutics for many years. Natural products have been selected during evolution to interact with biological targets and their high degree of chemical diversity make them attractive as lead structures for discovery of new drugs [4]. A number of plant-derived anticancer drugs have received FDA approval for marketing: taxol, vinblastine, vincristine, topotecan, irinotecan, etoposide and teniposide [5]. Antibiotics from Streptomyces species, including bleomycins, dactinomycin, mitomycin, and the anthracyclines daunomycin and doxorubicin are important anticancer agents [6]. More recently developed anticancer agents such as the Hsp90 inhibitor geldanamycin was also isolated from Streptomyces [7]. Marine organisms have also been used as source for the search of anticancer agents. Interesting compounds, including bryostatin (from the marine bryozan Bugula neritina), ecteinascidin (an alkaloid from the Carribian tunicate, Ecteinascidia turbinata) and dolastatin (from the sea hare), have been identified [8].

Although being the source of lead compounds for the majority of anticancer drugs approved by the Food and Drug Administration, natural products have largely been excluded from modern screening programs. We here used a high-throughput method for apoptosis detection [9] to screen a library of natural compounds using a human colon carcinoma cell line as screening target. One of the most interesting hits in this screen was thaspine, an alkaloid from the cortex of the South American tree *Croton lechleri*. We show that thaspine is a topoisomerase inhibitor which is active on cells overexpressing drug efflux transporters.

## Results

### Screening for natural products that induce apoptosis of colon carcinoma cells

**Figure 1:**
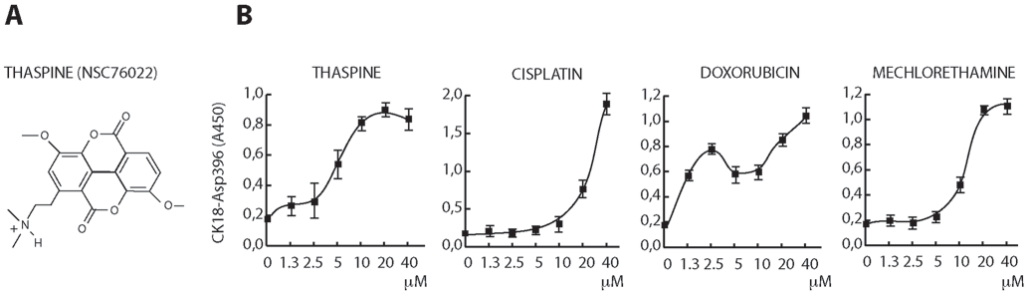
Induction of apoptosis by thaspine. (A) chemical structure of thaspine (NSC76022); (B) induction of caspase-cleaved CK18 by thaspine, cisplatin, doxorubicin and mechlorethamine in HCT116 colon carcinoma cells. Treatment was for 24 hours with the indicated concentrations of compounds. Cells were lysed and CK18-Asp396 was determined using the M30 CytoDeath ELISA. Results are shown with S.D. from triplicate determinations. Similar results (including the biphasic response to doxorubicin) were observed in independent experiments.

We used HCT116 colon carcinoma cells as target cells to screen for apoptosis-inducing agents present in NCI Natural Product Set (www.dtp.nci.nih.gov). Apoptosis was determined using a modification of the M30-Apoptosense® method [9] which specifically measures caspase-cleaved cytokeratin 18 formed in apoptotic cells. Activity in this assay is inhibited by the pan-caspase inhibitor zVAD-fmk [9]. The M30-Apoptosense® method is a useful screening tool since it measures the accumulation of the apoptotic product in cell cultures, leading to an integrative determination of apoptosis to the point of harvesting the cells. Using a compound concentration of 25 µM and an exposure time of 24 hours, 20 compounds were identified as inducing apoptosis above a preselected threshold value (Table 1). Molecular targets have been reported on 14 of these 20 compounds (Table 1). The alkaloid thaspine (taspine; NSC76022) was one of the remaining 6 compounds with unknown mechanism of action (Figure 1A). Thaspine is of interest since it is an alkaloid from Dragon's blood, a latex prepared from the cortex of the tree *Croton lechleri* and used by tribes in the Amazonas basin for medicinal purposes. Thaspine induced strong caspase-cleavage of cytokeratin-18 in HCT116 cells at a concentration of ∼10 µM (Fig. 1B). This concentration requirement is similar to that of other cancer therapeutic drugs such as cisplatin (∼20 µM), doxorubicin (∼3 µM) and mechlorethamine (∼20 µM) for induction of caspase activity of this cell line (Fig. 1B). Thaspine was also found to induce activation of caspase-3 at 10 and 16 hours (see below).

**Table 1:** Compounds in the NCI Natural Product Set that induce caspase cleavage of CK18 in HCT116 cells.

NSC number	Name	Target	Reference
5159	Chartreusin	DNA	[45]
19941	Champaca camphor		
26258	Rotenone	Electron transport	[46]
42076	Podophyllotoxin derivative	Microtubuli	[47]
62709	Streptonigrin	Topoisomerase II	[48]
76022	Thaspine		[10]
82151	Daunorubicin	Topoisomerase II	[49]
114344	Ascochitine	Protein phophatase	[50]
114568	Eupachlorin acetate		
122023	Valinomycin	K+ ionophore	[51]
135758	Piperazinedione	DNA alkylator	[52]
153858	Maytansine	Microtubuli	[53]
226080	Rapamycin	mTOR	[54]
234161	Deoxyliatrigramin		
250430			
285116	Siomycin A	FoxM1	[55]
287088	Physalin B	Proteasome	[56]
287456	Pacifenol		
332598	Rhizoxin	Microtubuli	[57]
333856	Tetrocarcin A	Bcl-2	[58]

### Thaspine induces apoptosis *in vivo*

**Figure 2:**
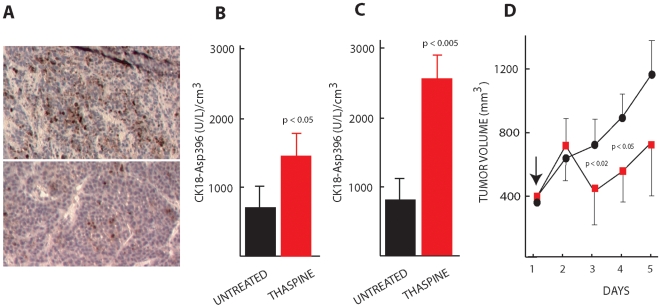
*In vivo* induction of tumor apoptosis by thaspine. (A) Induction of apoptosis in HCT116 tumors *in vivo*. SCID mice were injected with 10 mg/kg thaspine. Mice were sacrificed after 48 hours and tumors stained for active caspase-3 (upper panel: thaspine treated mice; lower panel: PBS injected mice). (B, C) SCID mice carrying HCT116 tumors (B) or FaDu head-neck carcinoma tumors (C) were injected with 10 mg/kg of thaspine or with PBS and the levels of human caspase-cleaved CK18 (CK18-Asp396) were determined in mouse plasma 48 hours after treatment using ELISA. The antibodies used to detect CK18-Asp396 do not bind mouse CK18. Four mice in each group; bars represent S.D. (D) SCID mice carrying FaDu tumors were treated with 10 mg/kg of thaspine (day 1, arrow) and tumor volume was calculated; black circles: untreated mice, red squares: treated mice (4 mice in each group). Error bars are presented as unidirectional for figure clarity. Animals were sacrificed when tumors were approximately 1 cm^3^ according to local animal welfare regulations. Statistical significance was calculated using Student's t-test.

Thaspine has previously been described to have anti-tumor activity in the mouse S180 sarcoma model [10]. To examine whether *in vivo* anti-tumor activity is associated with induction of apoptosis, SCID mice carrying HCT116 xenografts were treated with thaspine and tumor sections were stained with an antibody to active caspase-3. Positivity was observed in tumor tissue at 48 hours after treatment with 10 mg/kg thaspine (maximally tolerated dose) (Fig. 2A, top). We also utilized caspase-cleaved CK18 as a plasma biomarker for tumor apoptosis [11], [12]. When applied to human xenografts transplanted to mice, this method allows determination of tumor apoptosis independently of host toxicity (the antibodies used in the ELISA assay are species-specific and do not detect mouse caspase-cleaved CK18 [13]). We examined two different xenograft models using this assay, the HCT116 colon carcinoma used for screening and the FaDu head-neck carcinoma model. In order to mimic a clinical trial situation of advanced disease, tumors were allowed to grow to a size of ∼400 mm^3^ and then treated with a single injection of thaspine. Increases in CK18-Asp396 were observed 48 hours after injection of thaspine in both models (Figure 2B, C). Apoptosis was paralleled by a significant, but transient, reduction of tumor size in the FaDu model (Figure 2D). We conclude that thaspine is capable of inducing tumor apoptosis *in viv*o.

### Thaspine induces the mitochondrial apoptosis pathway

**Figure 3:**
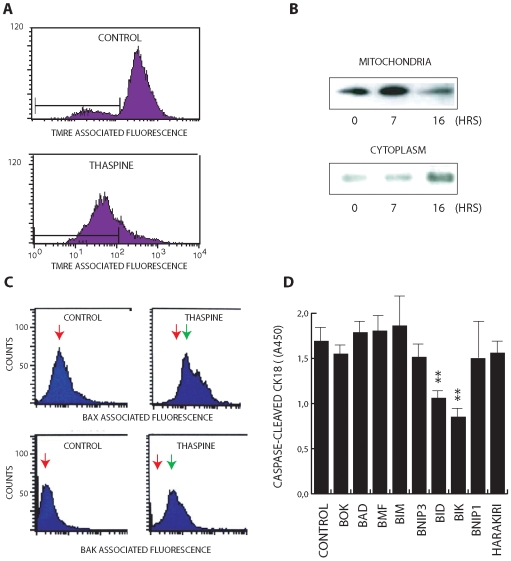
Induction of the mitochondrial apoptosis pathway by thaspine. (A) loss of mitochondrial membrane potential in thaspine-treated HCT116 cells. Control and thaspine-treated cells (10 µM, 18 hours) were stained with tetramethyl-rhodamine ethyl ester (TMRE) and fluorescence was measured by flow cytometry; (B) Release of cytochrome c to the cytosol of thaspine-treated cells. Cells were treated with 10 µM thaspine for 7 or 16 hours. Cytochrome c was quantified in mitochondrial and cytosolic fractions by Western blotting. (C) thaspine induces the active conformation of Bak and Bax. Cells were treated with 10 µM thaspine, fixed and stained with conformation-specific antibodies to Bak and Bax. Note induction of the active conformation of both molecules by thaspine (*red arrows*: immunofluorescent signal in untreated cells; *green arrows*: immunofluorescent signal in drug-treated cells). (D) Inhibition of thaspine-induced apoptosis by siRNA to Bik and Bid. Cells were transfected with siRNAs in triplicate 96 well plates and treated with thaspine or solvent (see Material and Methods). After 24 hours the levels of caspase-cleaved CK18 were determined using the M30 CytoDeath ELISA.

Most forms of cancer therapeutics induce the mitochondrial pathway of apoptosis [3]. This pathway is associated with opening of the mitochondrial permeability transition pore [14]. We examined whether thaspine induced a decrease in HCT116 mitochondrial membrane potential (Δψ_M_) using the fluorescent probe tetramethyl-rhodamine ethyl ester (TMRE). Mitochondria in thaspine-treated cells underwent a shift to lower Δψ_M_ values (Figure 3A). A hallmark of the mitochondrial apoptosis pathway is release of cytochrome c from mitochondria to the cytosol. Thaspine was found to induce a decrease in the levels of mitochondrial cytochrome c and an increase of the levels in the cytosol (Figure 3B). The Bcl-2 family proteins Bak and Bax are key regulators of the mitochondrial apoptosis pathway [15]. During apoptosis, the conformation of these proteins is altered. Experiments using conformation-specific antibodies showed that thaspine induce conformational activation of both Bak and Bax (Figure 3C). BH3-only proteins antagonize the pro-survival function of Bcl-2 proteins [16] or may activate pro-apoptotic Bak/Bax [17]. We used an siRNA approach to examine whether any particular BH3-only proteins were required for thapsin-induced apoptosis. Transfection with 9 different siRNA pools showed that Bid and Bik siRNA significantly reduced thaspin-induced cytokeratin 18 caspase-cleavage in HCT116 cells (Fig. 3D), suggesting that these proteins are regulators of apoptosis elicited by this compound.

### Thaspine is a topoisomerase inhibito

**Figure 4:**
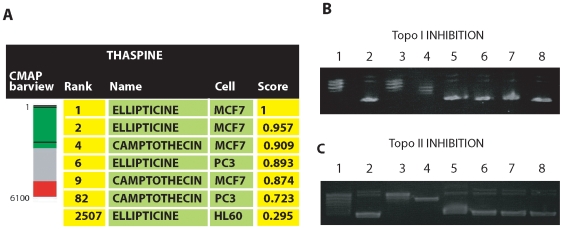
Thaspine is a topoisomerase inhibitor. (A) Connectivity Map (CMAP) results after treatment with thaspine. The bar view is constructed from 6100 horizontal lines, each representing a single treatment and ordered according to their corresponding enrichment to the query signatures generated after thaspine treatment. Black horizontal lines in the barview represent the individual instances for ellipticine and camptothecin when the thaspine expression signature was used as query signature. Score according to the CMAP database; (B) inhibition of topoisomerase I activity: 1: plasmid +5U topoisomerase I; 2: plasmid; 3: plasmid +5U topoisomerase I+DMSO (1 µm); 4. Marker for nicked plasmid; 5: plasmid +5U topoisomerase I +50 µM thaspine; 6: plasmid +5U topoisomerase I +25 µM thaspine; 7: plasmid +5U topoisomerase I +10 µM thaspine; 8: plasmid + DMSO (1 µm). Topoisomerase I primarily induced nicked plasmid DNA, note the inhibition by thaspine. (C) inhibition of topoisomerase II activity: 1: plasmid +15U topoisomerase II; 2: plasmid; 3: plasmid +15U topoisomerase II + DMSO (1 µm); 4. Marker for nicked plasmid; 5: plasmid +15U topoisomerase II +50 µM thaspine; 6: plasmid +15U topoisomerase II +25 µM thaspine; 7: plasmid +15U topoisomerase II +10 µM thaspine; 8: plasmid + DMSO (1 µm). Topoisomerase II primarily induced nicked plasmid DNA, note the inhibition by thaspine.

The molecular target of thaspine is not known. To generate hypotheses regarding the mechanism of action, we used the Connectivity Map (CMAP) [18], which is a large compendium of gene expression signatures from drug-treated cell lines. We analyzed the drug-induced gene expression changes compared to vehicle control in the breast cancer cell line MCF-7, since all of the 1,309 compounds in CMAP have been tested on this cell line. The gene expression response to thaspine was similar to that of ellipticine, a known topoisomerase II inhibitor, and similar to the response to the topoisomerase I inhibitor camptothecin (Figure 4A).

Topoisomerase inhibition was tested using *in vitro* enzyme assays. The results showed that thaspine inhibits both topoisomerase I and II activity at the apoptotic concentration (10 µM) (Figure 4B, C). Furthermore, thaspine was found to have a reduced cytotoxic effect on the viability on CEM/VM-1, a cell line selected for resistance to the topoisomerase II inhibitor teniposide compared to the parental cell line CCRF-CEM (Table 2). CEM/VM-1 harbors a mutated topoisomerase II gene which mediates a specific resistance to topoisomerase II inhibitors, but not general multidrug resistance [19], [20]. CCRF-CEM are not resistant to camptothecin [21]. The resistance to thaspine was not as pronounced as seen for etoposide, known to be a non-intercalating topoisomerase II inhibitor, but well in line with the intercalating topoisomerase inhibitors doxorubicin and mitoxantrone. These data further suggest that thaspine is a topoisomerase inhibitor.

**Table 2:** Influence of resistance mechanisms on cytotoxic potency of thaspine and standard topoisomerase inhibitors.

	Topo II associated	P-gp associated	MRP associated
Parental cell line	CCRF-CEM	RPMI 8226	NCI-H69
Resistant cell line	CEM/VM-1	8226/Dox40	H69AR
Thaspine	4.8	1.8	1.1
Doxorubicin	3.9	87.0	13.5
Etoposide	12.0	13.0	61.6
Mitoxantrone	4.6	10.4	1.7

The resistance factor was defined as the IC_50_ value in the resistant subline divided by that in its parental cell line. The IC_50_ of thaspine on the parental cell lines were between 1–3 µM. Topo II, Topoisomerase II; P-gp, P-glycoprotein (ABCB1); MRP, multidrug resistance-associated protein (ABCC1). For details on cell lines and calculations of resistance factors, see [22]. CCRF-CEM are not resistant to camptothecin [21], which was therefore not included. The experiments were repeated twice.

Thaspine induced an accumulation of HCT116 cells in the S and G2/M phases of the cell cycle (Table 3). For comparison, the topoisomerase II inhibitor etoposide induced G2/M accumulation, whereas camptothecin induced some S-phase arrest (Table 3).

**Table 3:** Effects of thaspine and reference compounds on the HCT116 cell cycle.

	G1 (percent)	S (percent)	G2 (percent)
Control	37.8+6.1	37.0+0.4	25.1+5.8
Thaspine	13.5+2.7	49.6+2.2	36.9+2.5
Etoposide	11.1+2..9	42.1+1.2	46.7+3.4
Camptothecin	35.3+1.1	50.4+1.7	14.3+1.9

### Thaspine cytotoxicity is only weakly affected by PgP or MRP overexpression

Anticancer drugs are often substrates of membrane-associated drug efflux transporters. The topoisomerase II inhibitor doxorubicin is a substrate of P-glycoprotein (Pgp, ABCB1) and etoposide is a substrate of the multidrug resistance-associated protein (MRP, ABCC1). We found that two cell lines which are strongly resistant to etoposide and/or doxorubicin due to PgP or MRP overexpressio [22] were <2-fold resistant to thaspine compared to parental cells (Table 2).

### Thaspine induces apoptosis in multicellular spheroids

**Figure 5:**
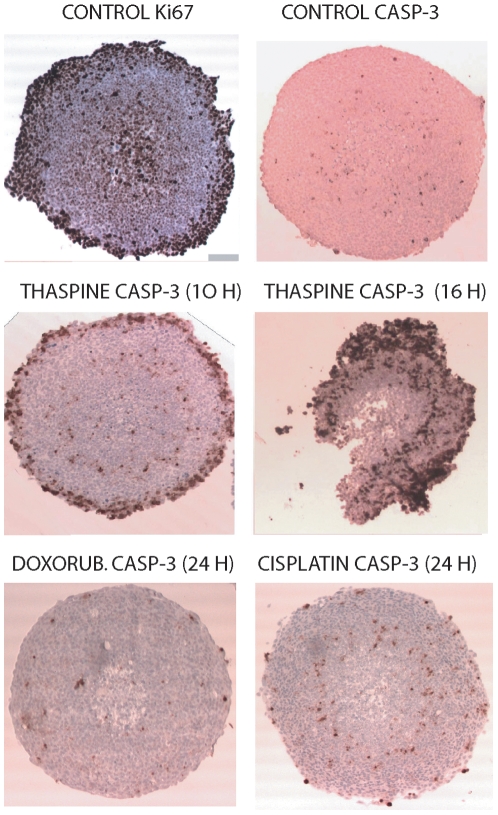
Thaspine induces wide-spread activation of caspase-3 in spheroids. HCT116 spheroids with homogeneous diameters were formed using the hanging drop technique as described [40]. Five days after formation spheroids were compact and contained proliferating cells only in the surface layers (Ki67 staining, top left). Spheroids were treated with drugs for the times indicated, fixed, sectioned and stained for active caspase-3. Thaspine was used at 20 µM, doxorubicin at 20 µM, and cisplatin at 40 µM.

Multicellular spheroids (MCS) are known to better mimic human solid tumor tissue than 2-D monolayer cultures. Many clinically used anticancer drugs show limited potency on MCS, a phenomenon believed to reflect their limited activity on solid tumors [23], [24]. To investigate whether thaspine induces apoptosis of MCS, spheroids were formed from HCT116 and used after 5 days of incubation. At this point in time, cell proliferation in the MCS was largely confined to peripheral cell layers (Fig. 5, top left, staining for Ki67) and some spontaneous apoptosis was observed in deeper cell layers (Fig. 5, top right, staining for active caspase-3). Following drug treatment, MCS were fixed, sectioned and stained for active caspase-3. Activation of caspase-3 was observed in MCS after 10 hours of treatment with thaspine, and wide-spread activation after 16 hours of treatment (Fig. 5). Cells in the central portions of MCS did not stain positive for active caspase-3 even at the time of spheroid disintegration. To determine cell survival, spheroids were trypsinized and cells were plated at low density to determine clonogenicity. Clonogenic survival of cells from spheroids treated with 10 µM thaspine was 0.006% of cells from control spheroids. These data show that thaspine treatment was able to kill the cells in the spheroid cores, but that cell death was not by apoptosis. Cisplatin and doxorubicin did not induce wide-spread apoptosis in HCT116 MCS (Fig. 5).

## Discussion

We here screened a collection of natural products for their capacity to induce apoptosis of colon carcinoma cells. Natural products are known to have a high chemical diversity [4], a necessity for drug discovery in the oncology field [25]. This approach lead to the identification of 20 agents that induced strong increases in the levels of caspase-cleaved cytokeratin 18 in colon carcinoma cells. Several of these compounds are well known to have anti-tumor activity (e.g. podophyllotoxin, daunorubicin, maytansine, rapamycin and rhizoxin). Of the remaining compounds we noted thaspine (taspine), an alkaloid present in the cortex of the South American tree *Croton lechleri*. Thaspine is of interest since *Croton lechleri* is used in traditional medicine. A red latex, Dragon's blood, is extracted from the tree cortex and used by tribes of the Amazonian basin for several purposes, including wound healing, as an anti-inflammatory agent, and to treat cancer [26], [27]. Thaspine was previously reported to be cytotoxic [26], [28], [29], anti-angiogenic [30], and to have antitumor activity [28]. Consistent with these previous reports, we found that thaspine treatment induced caspase activation in tumor tissue and release of human caspase-cleaved CK18 from tumor cells into the blood of SCID mice.

Our connectivity map analysis showed that thaspine induced a similar gene expression pattern as the topoisomerase inhibitors ellipticine and camptothecin. Direct measurements of enzyme activity confirmed that both topoisomerase I and II were inhibited by relevant concentrations of thaspine. Furthermore, CEM/VM-1 cells, which express a mutated form of topoisomerase II resistant to inhibitors of this enzyme [19], [20], showed increased resistance to thaspine. Topoisomerases are enzymes which have important roles in DNA metabolism by adjusting the number of supercoils in the DNA molecule - a key requirement for transcription and replication. Topoisomerase I is capable of introducing single strand breaks in DNA, while topoisomerase II can break both strands. A variety of clinically used anticancer drugs inhibit the action of topoisomerase I [31], [32] or topoisomerase II [33]. The topoisomerase I inhibitors topotecan and irinotecan are among the most effective drugs used to treat colorectal, small cell lung and ovarian cancer [31]. Topotecan and irinotecan are chemically unstable and large efforts are being made to develop improved compounds [32]. A large number of compounds have been described to inhibit topoisomerase II, including the important clinical agents doxorubicin/adriamycin and etoposide [33]. A limited number of agents can inhibit both enzymes and may have strong antitumor activity [34]. Some agents such as intoplicine, the acridine XR5000 (DACA) bind to DNA by intercalation [35], others are physically linked inhibitors of topoisomerase I and topoisomerase II.

Drug resistance is the most important cause of cancer treatment failure and represents a major challenge to the treatment and eradication of cancer. Drug resistance is known to be multifactorial. One important mechanism of resistance to clinically used DNA damaging anticancer drugs is the expression of ABC transporters such as Pgp and MRP [36]. Thaspine cytotoxicity was only marginally affected by overexpression of the P-glycoprotein (Pgp, ABCB1) or the multidrug resistance-associated protein (MRP, ABCC1). Another mechanism of resistance of solid tumors to anticancer drugs is multicellular-mediated resistance [37]. This form of resistance has been found to influence the effect of adriamycin on solid tumors, partly due to limited drug penetration into the tumor parenchyme [38], [39]. Interestingly, thaspine was found to induce wide-spread apoptosis of multicellular spheroids. This property is interesting considering that many clinically used anticancer drugs show limited potency on spheroids, possibly reflecting their limited activity on solid tumors [23], [24]. The therapeutical potential of thaspine on solid tumors is therefore interesting to examine. Such studies require optimization of drug formulation (thaspine is hydrophobic; XlogP  = 2.8) and evaluation of how thaspine should be combined with other drugs.

Only a fraction of the diversity of the biosphere has been tested for biological activity. The approach to screen chemically diverse drug libraries for apoptosis-inducing compounds and to use the Connectivity Map resource [18] for unveiling the mechanisms of action of the compounds identified is reasonably straight-forward. We believe that this approach may be effective in anticancer drug discovery.

## Materials and Methods

### Compounds

The NCI Natural Product Set was obtained from the Developmental Therapeutics Program of the US National Cancer Institute (http://www.dtp.nci.nih.gov).

### Cell culture and screening

HCT116 colon carcinoma cells (from the American Type Culture Collection, ATCC) were maintained in McCoy's 5A modified medium/10% fetal calf serum at 37°C in 5% CO_2_. Multicellular spheroids were formed from HCT116 cells as described [40]. For description and of cell lines MCF-7, CCRF-CEM, CEM/VM1, RPMI 8226, NCI-H69, H69AR see [22]. Cells were seeded in 96 well plates at 10,000 cells per well. Compounds were added to a concentration of 25 µM in a final concentration of 1% DMSO; control wells received 1% DMSO only. After 24 hours of drug treatment, NP40 was added to the culture medium to 0.1% to extract caspase-cleaved CK18 from cells and to include material released to the medium from dead cells. Caspase-cleaved cytokeratin-18 (CK18-Asp396) was determined using 25 µl medium/extract using the M30 CytoDeath ELISA assay (a variant of the M30-Apoptosense® ELISA [9] developed for *in vitro* use (Peviva AB, Bromma, Sweden)). Signals were factorised to percent of a staurosporine reference (quadruple wells used on each plate at 1 µM) and compounds that induced a signal >60% of staurosporine were selected for study.

Resistance mediated by efflux proteins and mutated topoisomerase II was evaluated using the fluorometric microculture cytotoxicity assay (FMCA) [41]. The resistance factor was defined as the IC_50_ value in the resistant subline divided by that in its parental cell line [42].

#### siRNA transfection

HCT116 cells were transfected in 96 well plates with siRNAs to BH-3 only proteins using HiPerfect transfection reagents as recommended by the manufacturer (all reagents from QIAGEN, Hilden, Germany). Pools of 3 different siRNAs were used (final concentration 25 nM) for triplicate wells for each siRNA pool. After 54 hours of incubation, thaspine or solvent was added to the wells of the 96 well plates and incubation was continued for 18 hours. CK18-Asp396 was then determined using the M30 CytoDeath ELISA as described above.

### Immunological assays

Conformational changes resulting in exposure of inaccessible N-terminal epitopes of Bak and Bax were determined using flow cytometry [43]. Conformation-specific antibody to Bak was obtained from Oncogene Research Products) (AM03, clone TC100) and to Bax from BD Bioscieces PharMingen (clone 6A7; San Diego, CA). The increases in accessibility of the epitopes was monitored using the FL1 channel of a FACScalibur flow cytometer (BD, Franklin Lakes, NJ) as previously described [44]. Tumor or spheroid sections were deparaffinized with xylene, rehydrated and microwaved and then incubated over-night with the primary antibodies diluted in 1% (wt/vol) bovine serum albumin and visualized by standard avidin–biotin–peroxidase complex technique (Vector Laboratories, Burlingame, CA, USA). Counterstaining was performed with Mayer's haematoxylin. Antibody against active caspase-3 was from Pharmingen (used 1∶50) and antibody to Ki67 (MIB-1) was from Immunotech SA, Marseille, France (used at 1∶150).

### Mitochondrial Membrane Permeability Transition

Loss of mitochondrial membrane potential was monitored using tetramethyl-rhodamine ethyl ester (TMRE) from Molecular Probes (Eugene, OR). Fluorescence was measured by flow cytometry.

### Assay of cytochrome c release

HCT116 cells were treated with 10 µM thaspine and harvested at 7 or 16 hours. After washing with PBS, cells were resuspended in 500 µL (10 mM NaCl, 1.5 mM CaCl2, 10 mM Tris, pH 7.5) and incubated for 20 min on an ice bath. After passing 10 times through a 40 gauge needle, 400 µL mitochondrial buffer (210 mM mannitol, 70 mM sucrose, 20 mM Hepes-KOH, pH 7.5, and 1 mM EDTA containing 0.45% bovine serum albumin (BSA)) was added and nuclei were pelleted by centrifugation for 1000 xg for 10 min at +4°C. The supernatant was trasferred to a new tube and centrifuged for 30 min at maximal speed in an Eppendorf centrifuge at +4°C. Supernatant and pellet fractions were analysed by Western blotting (cytochrome c antibody ab33484 from Abcam used at 1∶1000).

### Connectivity Map

The Connectivity Map (CMAP) (www.broad.mit.edu/cmap) build 02 contains genome-wide expression data for 1300 compounds (6100 instances, including replicates, different doses and cell lines). We followed the original protocol using MCF-7 breast cancer cells as described by Lamb *et al* [18]. Briefly, cells were seeded in a 6-well plate at a density of 0.4×10^6^ cells per well. Cells were left to attach for 24 h, followed by exposure to thaspine at a final concentration of 10 µM, or to vehicle control (DMSO). After 6 h treatment, the cells were washed with PBS and total RNA was prepared using RNeasy miniprep kit (Qiagen, Chatsworth, CA). Starting from two micrograms of total RNA, gene expression analysis was performed using Genome U133 Plus 2.0 Arrays according to the GeneChip Expression Analysis Technical Manual (Rev. 5, Affymetrix Inc., Santa Clara, CA). Raw data was normalized with MAS5 (Affymetrix) and gene expression ratios for drug treated *vs.* vehicle control cells were calculated to generate lists of regulated genes. Filter criteria were present call for all genes in the treated cell line and an expression cut-off of at least 100 arbitrary expression units. Only probes present on HG U133A were used, for CMAP compatibility. The 40 most up and down regulated genes (i.e. probes) were uploaded and compared to the 6100 instances in the CMAP database, to retrieve a ranked compound list. Raw and normalized expression data have been deposited at Gene Expression Omnibus (http://www.ncbi.nlm.nih.gov/geo/) with accession number GSE13124.

### Topoisomerase enzyme assays

Tests of topoisomerase inhibition were performed using kits from TopoGen Inc. (Port Orange, FLA) according to the instructions of the manufacturer. All incubations were performed in the presence of solvent (DMSO) for 30 minutes at 37°C followed by agarose gel electrophoresis in the absence of ethidium bromide. Gels were stained with ethidium bromide, washed and photographed.

### Treatment of mouse xenografts and determination of caspase-cleaved CK18 in mouse plasma

HCT116 or FaDu tumors were grown as subcutaneous xenografts in SCID mice (4 mice in each group; each injected with 10^6^ cells). When tumors had grown to a size of approximately 400 mm^3^ (somewhat larger than the generally used size; a larger tumor size leads to better sensitivity using the biomarker assay), mice were injected with thaspine subcutaneously and tumor size measured daily. The dose used (10 mg/kg) was tolerated by the mice (toxicity was observed at 20 mg/kg). FaDu cells express high levels of CK18 which is released to the extracellular compartment and into the blood from dying cells. Mice were sacrificed 48 hours after injection of thaspine and EDTA-plasma was collected. Caspase-cleaved CK18 (CK18-Asp396) was measured in 12.5 µL of plasma using the M30-Apoptosense® assay (Peviva AB, Bromma). Each sample was mixed with 0.4 µL of heterophilic blocking reagent (HBR-Plus purified, part#3KC579; Scantibodies laboratory Inc, Santee CA, USA). Animal experiments were conducted in full accordance with Swedish governmental statutory regulations on animal welfare under permission from ethical committees (permit N295/06 Stockholms norra djurförsöksetiska nämnd).
